# Molecular mechanism of hepatitis B virus (HBV) on suppression of raf kinase inhibitor protein (RKIP) expression

**DOI:** 10.18632/oncotarget.13586

**Published:** 2016-11-25

**Authors:** Xiao-Ke Cheng, Guo-Zheng Yu, Xiao-Dong Li, Xue-Qun Ren

**Affiliations:** ^1^ Center for Evidence-Based Medicine, Huaihe Hospital of Henan University, Kaifeng 475000, Henan Province, China; ^2^ Department of General Surgery, Huangshi Central Hospital, Affiliated Hospital of Hubei Polytechnic University, Huangshi 435000, Hubei Province, China; ^3^ Department of Urology, Huaihe Hospital of Henan University, Kaifeng 475000, Henan Province, China; ^4^ Department of General Surgery, Huaihe Hospital of Henan University, Kaifeng 475000, Henan Province, China

**Keywords:** RKIP, HBV, liver cancer, AP1protein, promoter methylation

## Abstract

Raf kinase inhibitor protein (RKIP) has been shown to be a suppressor of the mitogen-activated protein kinase pathway and is reported to be involved in human malignancy. However, the molecular mechanism of hepatitis B virus (HBV) in regulating RKIP expression is not yet clarified. In this study, we compared RKIP expression in 107 pairs of matched liver cancer and adjacent non-cancerous liver tissues. Among seven HBV-encoded proteins, we found HBV X (HBX) protein could significantly inhibit the expression level of RKIP, indicating that HBV could suppress RKIP expression through regulating HBX. To further elucidate the mechanism, analyses on transcriptional regulation and promoter methylation inhibition were conducted in Huh7 cells. Our results showed that HBX can interact with AP1 protein to inhibit the RKIP transcription. Moreover, we observed that the promoter methylation level of RKIP could be enhanced by HBV. In conclusion, our study revealed that RKIP could act as a molecular marker for HBV-infected liver cancer, but had no tumor-suppressing effect.

## INTRODUCTION

Hepatitis B virus (HBV) is a section of non-cytopathic and double-stranded DNA virus that causes acute and chronic liver diseases. HBV infection is a major public health threat to human health, and there are about one million people dying of liver diseases caused by HBV infection, such as liver failure, cirrhosis, and liver cancer, every year around the world [1]. Globally, about 75% of hepatocellular carcinoma (HCC) cases are developed from HBV infection [2]. After Lacent first reported the relationship between HBV and HCC in 1970, a number of other studies further confirmed their conclusion. Nevertheless, the mechanism through which HBV infection results in liver cancer remains largely unclear.

Studies have demonstrated that hepatitis B virus X (HBX) plays an important role in HBV-mediated HCC. HBX protein is a multifunctional regulatory protein, which can participate in a variety of cellular activities such as intracellular signal transduction, transcription, cell cycle regulation, cell apoptosis and autophagy through interacting with different host cytokines.

DNA methylation is one of the important layers of DNA base modification. It has been revealed that methylation of CpG islands within promoter and/ or 5′-regulatory regions could frequently lead to gene transcription silencing [3]. Methylation of DNA has shown close correlation with tumorigenesis. Studies have also indicated that alteration in DNA methylation status may be involved in inactivation of tumor suppressor genes [4].

As a recently-discovered tumor suppressor, the Raf kinase inhibitor protein (RKIP) is a member of the phosphatidylethanolamine-binding protein superfamily, which participates in tumor growth, transformation and differentiation [5]. Functional studies have shown that promoter methylation of RKIP is more frequently associated with decreased RKIP expression compared to genetic mutations or loss of heterozygosity. Furthermore, RKIP has been reported to play important roles in numerous human malignancies [6–8].

## RESULTS

### RKIP expression in tumor tissues

**Figure 1:**
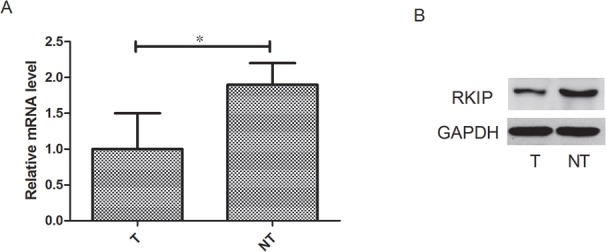
RKIP expression discriminates between live cancer tissues and paired non-cancerous tissues **A.** RKIP mRNA expression was significantly reduced in tumor tissues compared with normal adjacent tissues. **B.** RKIP protein expression was significantly reduced in tumor tissues compared with normal adjacent tissues. n=107, *P<0.05.

We measured the RKIP mRNA expression by real-time RT–PCR and protein expression by western blot analysis in liver cancer tissues and paired non-cancerous tissues. Compared with the non-cancerous controls, the mRNA expression level of RKIP was significantly reduced by 47% in tumor tissues (*P*<0.05, Figure 1A), and the protein level was also obviously reduced in tumor tissues (*P*<0.05, Figure 1B). The results showed that RKIP expression was considerably diminished in tumor tissues, suggesting decreased or absence of RKIP expression might contribute to hepatocarcinogenesis.

### Correlation of HBV and HBX with the promoter activity of RKIP

**Figure 2:**
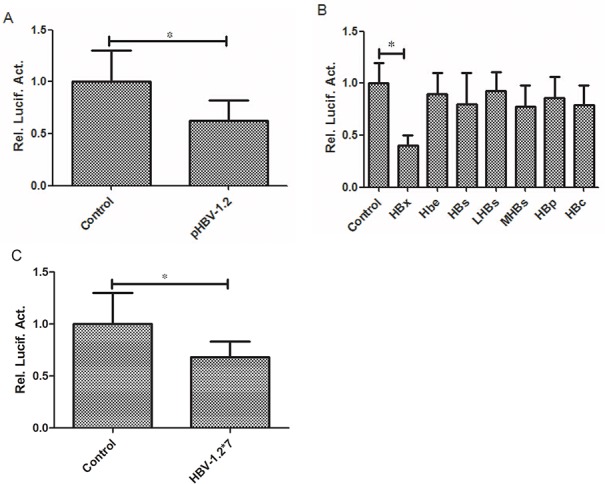
HBV-1.2 and HBX inhibit the promoter activity of RKIP **A.** HBV-1.2 significantly suppressed RKIP promoter activity (P<0.05). **B.** Among the seven proteins of HBV co-transfected with PRIK promoter in Huh7 cells, only HBX protein significantly inhibited RKIP promoter activity, other proteins showed no obvious inhibition effects. **C.** In Huh7 cells, HBV-1.2*7 had no obvious effect on RKIP promoter activity.

The Huh7 cells were co-transfected with plasmid (pCMV HBV-1.2, pCMV-HBV-1.2*7, pCMV HBX, Hbe, HBs, LHBs, MHBs, HBP and HBc) and the promoter pGL3-RKIP. The luciferase activity was measured in each sample 48 hours after transfection. pCMV-pblue and pCMV-flag-2b were used as controls. Our results showed that HBV-1.2 significantly suppressed RKIP luciferase activity by 37% (*P*<0.05, Figure 2A), and among the seven proteins of HBV, only HBX protein significantly inhibited RKIP luciferase activity by 32% (*P*<0.05, Figure 2B). However, HBV-1.2*7 had no obvious effect on RKIP luciferase activity (*P*>0.05, Figure 2C).

### Impact of HBV and HBX on the mRNA and protein expression levels of RKIP

**Figure 3:**
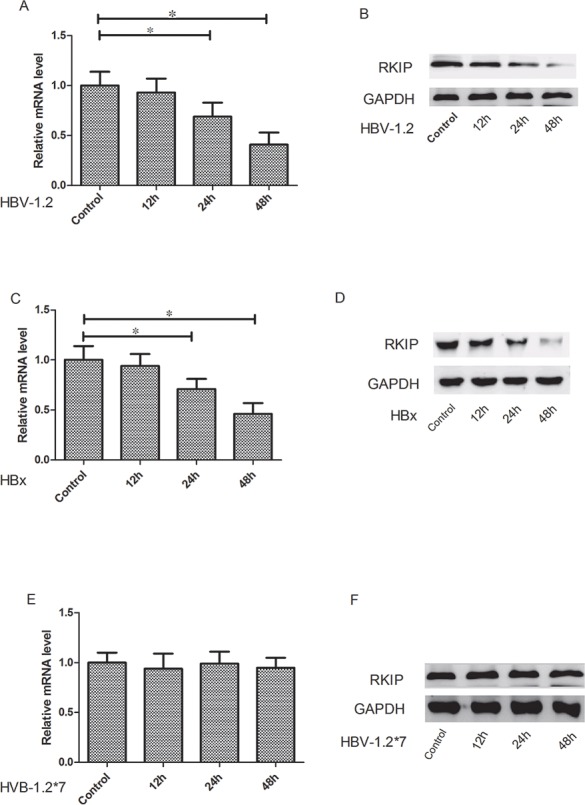
HBV-1.2 and HBX inhibit the mRNA and protein expression levels of RKIP The Huh7 cells were transfected with pCMV-HBV-1.2, pCMV-HBV-1.2*7 and pCMV-HBX, respectively. Then the RKIP mRNA and protein expression levels were detected at different time points (0, 12, 24 and 48 h). Cells transfected at 0 h was used as control. **A, B.** HBV-1.2 obviously inhibited the RKIP mRNA and protein expressions after transfection at 24h and 48h. **C, D.** HBX significantly inhibited the expression of RKIP mRNA and protein expressions at 24h and 48h. **E, F.** HBV-1.2*7 did not change RKIP mRNA and protein expression significantly (P>0.05).

The Huh7 cells were transfected with pCMV-HBV-1.2, pCMV-HBV-1.2*7 and pCMV-HBX, respectively. Then the RKIP mRNA and protein expression levels were detected at different time points (0, 12, 24 and 48 hours). Cells transfected at 0 h was used as the controls. Our experiment showed that HBV-1.2 obviously inhibited the RKIP mRNA and protein expressions after transfection at 24h (*P*<0.05) and 48h (*P*<0.05) (Figure 3A, 3B). Similarly, HBX significantly inhibited the expression of RKIP mRNA and protein expressions at 24h (*P*<0.05) and 48h (*P*<0.05) (Figure 3C, 3D). However, HBV-1.2*7 did not change RKIP mRNA expression significantly (*P*>0.05, Figure 3E, 3F).

### Effects of AP1 in regulating the promoter activity of RKIP

**Figure 4:**
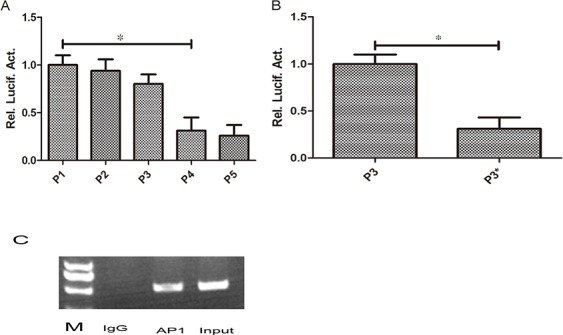
AP1 play an important role in regulating the promoter activity of RKIP **A.** The full length promoter of RKIP (P1) and a series of truncations (P2, P3, P4, P5) were transiently transfected into Huh7 cells, then luciferase activity was detected after 48 hours. We found the promoter activity of RKIP was decreased significantly after truncation of nucleotides −90to+3 segment in P3 (P4) (P<0.05). **B.** Then we transiently transfected AP1 mutant sites in Huh7 cells and detected the promoter activity after 48 hours, we found that the promoter activity was decreased significantly in P3 mutant group compared to control (P<0.05). **C.** We used CHIP experiment to verify the AP1 binding site, clear bands can be seen in input and AP1 lanes. No band was found in IgG lane.

The full length promoter of pGL3-RKIP and a series of truncations (P2, P3, P4, P5) were transiently transfected into Huh7 cells, then luciferase activity was detected after 48 hours. We found the promoter activity of RKIP was decreased by 61% after truncation of nucleotides-90to+3 segment in P3 (*P*<0.05, Figure 4A), suggesting it might be the core area of RKIP promoter. We then analyzed the binding sites of this segment sequence using genomatix software (http://www.genomatix.de/) combined with relevant literature. It was hypothesized that the AP1 sites might play an important role. Then we transiently transfected AP1 mutant sites into Huh7 cells and detected the promoter activity after 48 hours, we found that the promoter activity was decreased by 60% (*P*<0.05, Figure 4B). Furthermore, we used CHIP experiment to verify whether there were AP1 binding sites located in the RKIP promoter. Our results showed that the AP1 binding site sequence could be clearly detected in AP1 antibody precipitated complexes, suggesting the AP1 was combined with RKIP promoter (Figure 4C).

### Effects of HBV on RKIP promoter methylation

**Figure 5:**
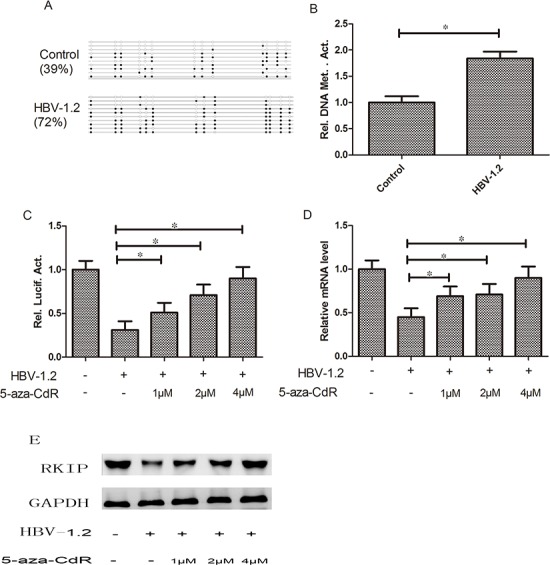
HBV can affect RKIP expression through regulating the promoter methylation status **A, B.** Cells with HBV-1.2 transfection exhibited higher RKIP promoter methylation level than the control group (P<0.05). **C, D, E.** The promoter activity of RKIP and its mRNA and protein expression levels were increased significantly by increased amounts of 5-aza-CdR treatment, respectively (P< 0.05).

The detection results showed that cells with HBV-1.2 transfection exhibited higher RKIP promoter methylation level by nearly 2-fold (*P*<0.05) than the control group (Figure 5A, 5B). To further investigate the effects of HBV-1.2 on RKIP promoter methylation, we transiently transfected promoter of pGL3-RKIP and HBV-1.2 into Huh7 cells, and treated with different amounts of 5-aza-CdR (1, 2 and 4 μM). Promoter luciferase activity was increased significantly by 1.6-fold, 2.3-fold and 2.9-fold after treatment with 1, 2 and 4μM of 5-aza-CdR treatment, respectively (both *P*<0.05, Figure 5C). RKIP mRNA expression level was increased significantly by 1.5-fold, 1.6-fold and 2.0-fold after treatment with 1, 2 and 4μM of 5-aza-CdR treatment, respectively (both *P*<0.05, Figure 5D). In agreement, the expression of RKIP at the protein level was also increased (Figure 5E). All these results demonstrated that the methylation inhibitor of 5-aza-CdR could play a part in the effects of HBV on RKIP, and we hypothesized that HBV could inhibit the expression of RKIP through its promoter methylation.

### Interaction between HBX and AP1

**Figure 6:**
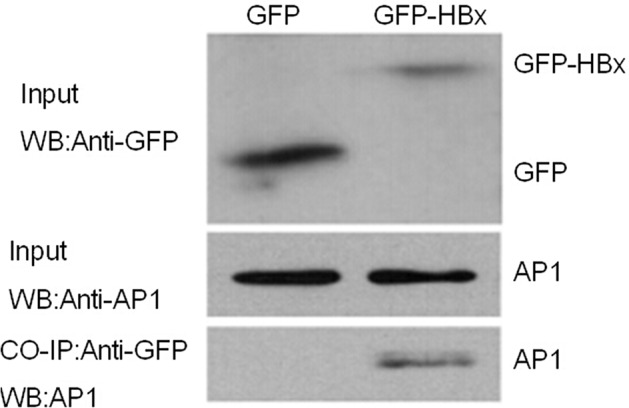
HBX and AP1 are mutually interacted with each other GFP-HBx and GFP were transiently transfected into Huh7 cells. As shown in the figure of Chip experiment, GFP expression can be detected and the AP1 expression were similar in two groups. AP1 can only be detected in precipitated complex of GFP-HBx, not in GFP group.

To investigate the possible interactive effects between HBX and AP1, we cloned HBx into plasmid eGFP-N3, making it more convenient for detecting the expression as well as strengthening its stability. Then GFP-HBx plasmid was transfected into Huh7 cells, and a rabbit polyclonal antibody against GFP was used to precipitate the complexes of GFP-HBx and endogenous AP1. Finally, a monoclonal antibody against AP1 was used to detect the complex. Our results showed that GFP empty vector and GFP-HBx were respectively expressed in cells, and AP1 input was also appropriate. However, AP1 was only detected in precipitated complex with HBx expression, suggesting HBx and AP1 protein had interactions therebetween (Figure 6).

## DISCUSSION

Being a member of the phosphatidylethanolamine-binding protein superfamily, RKIP is a cytosolic protein originally purified from bovine brain [9]. The Ras/Raf/ERK pathway has been so far regarded as one of the most active signaling pathway, and plays an important role in the cellular process of malignant transformation [10–11]. Recent studies have shown that RKIP is considered as a tumor metastasis suppressor gene that can inhibit the metastases of multiple human malignancies, such as malignant melanoma [12], breast cancer [13], colorectal cancer [14] and liver cancer [15].

DNA methylation is a way of chemical modification of genetic material in eukaryotes and is catalyzed by DNA methyltransferase (DNMT). Repetitive sequences of certain genes are hypermethylated in normal cells, which can prevent re-activation of the transcription factors and therefore help maintaining genomic stability [16–18]. When aberrant methylation occurs in somatic cells (demethylation of the genome), it can induce uncontrolled cell growth, and the changes in specific sites (promoter hypermethylation of tumor suppressor genes in CpG islands) can result in tumorigenesis. Aberrant DNA methylation is an epigenetic feature of tumor cells [19–21]. Since hypermethylation of tumor cells is usually localized in promoter regions of genes and can result in gene expression silencing, more attention should be paid on hypermethylation localized in tumors, including liver cancer. Studies have demonstrated that some viruses can cause promoter hypermethylation of tumor suppressor genes and affect their transcription and expression, which might lead to abnormal cell proliferation and then promote carcinogenesis [22].

Commonly, DNA methylation is irreversible. Relevant researches have issued that epigenetic drug such as 5-aza-2′-deoxycytidine (5-aza-CdR) can be used to reactivate genes silenced by aberrant DNA methylation. 5-aza-CdR is an analogue of deoxycytidine and can be incorporated into DNA synthesis. The incorporation is irreversible and therefore the ability of DNA to accept methyl group by DNA methyltransferases (DNMTs) is accepted. Meanwhile, DNMTs activity can be further reduced when covalent complexes are formed [23]. In our study, the 5-aza-CdR treatment significantly increased RKIP expression, implying that 5-aza-CdR inhibited the effect of HBV on RKIP promoter methylation.

Liver cancer is a rather common tumor that is easy to relapse and metastasize and is with high mortality and poor prognosis [24]. It has been reported that about 80% of liver cancer cases were attributable to HBV infection [25–26]. Thus, it is of great value to find out specific biomarkers for earlier diagnosis of liver cancer and new targets for treatment. This experiment was aimed to explore the mechanism of RKIP and HBV in the development of liver cancer and to provide a theoretical basis on clinical therapy and prognosis judgement through analyzing the expression and clinical characteristics of RKIP in primary tumor lesions. Our results uncovered that HBX protein could induce the aberrant promoter methylation of RKIP and lead to reduced RKIP expression, which were involved in the hepatocarcinogenesis and cancer development. The findings indicated that the expression of RKIP induced by the methylation inhibitor 5-aza-CdR could provide a potential guidance for treatment of hepatitis B caused-liver cancer. Generally, this study provided a theoretical basis for the clinical treatment of liver cancer caused by hepatitis B virus.

In the present study, we found that the RKIP expression was decreased significantly in HBV infected tumor tissues compared to normal adjacent tissues, suggesting the reduction or absence of RKIP expression was closely related to liver carcinogenesis. It was then verified in our in vitro experiment that the decreased expression of RKIP was affected by HBV virus. The reasons for insignificant effects of HBV on RIKP expression in normal adjacent tissues need to be further investigated.

## MATERIALS AND METHODS

### Patients and specimens

From September 2014 to January 2016, 107 pairs of matched liver cancer and adjacent non-cancerous liver tissues were collected from patients subjected to liver resection at the Huaihe Hospital of Henan University with their informed consent. All 107 patients were pathologically diagnosed as liver cancer cases with HBV infection for over 12 months. No patients received pre-operative radiotherapy or chemotherapy. Among these cases, 69 participants were men, and 38 were women. The median age of them was 54.61±10.13 years, with the age ranging between 32 and 81 years. This study was approved by the Huaihe Hospital of Henan University research ethics committee.

### Plasmid construction and reagents

The HBV plasmid carrying 1.2-fold length, replication-competent HBV genome (pCMV-HBV-1.2 subtype adw) and the same plasmid with a stop codon for amino acid 7 of HBx (pCMV-HBV-1.2*7) were obtained from the state key laboratory of virology of Wuhan University. Construction of the luciferase reporter vector (pGL3-Basic) (Promega Corporation) contained a 2017bp promoter construct of the RKIP gene, the promoter transcriptional region site (nucleotides-2000to+17), its truncated or site-specific mutants generated from human genomic DNA by polymerase chain reaction. The resulting construct was confirmed by DNA sequencing of RKIP. To study the role of HBX in the hypermethylation function, the effects of pCMV-HBV-1.2 and pCMV-HBV-1.2*7 on the expression of RKIP were compared. There were seven proteins in the HBV genome, including HBX, HBc, LHBs, MHBs, Hbe, HBP and SHBs. The plasmids were constructed using the pCMV-tag2b vector. To evaluate the predominant protein involved in the regulation of RKIP, these plasmids were each transfected and the expression of RKIP was subsequently analyzed. The plasmid pCMV-pblue and the promoter pGL3-flag-2b were constructed and then used as controls. The 5-aza-CdR and all antibodies against were purchased from Cayman (Ann Arbor, MI, USA).

### Cell culture

The human hepatoma cell-line Huh7 was obtained from the state key laboratory of virology of Wuhan University, and was characterized by mycoplasma detection, isozyme detection, DNA fingerprinting, and cell vitality detection. Huh7 was grown in Dulbecco's modified Eagle's medium (DMEM) supplemented with 10% (vol/vol) fetal bovine serum (Gibco, USA), 100 μg/ml streptomycin (Sigma, St. Louis, MO), and 100units/ml penicillin (Invitrogen Life Technologies) sulfate at 37°C in 5% CO_2_.

### Transfection

All transfections were performed using Sofast® reagent (sunma, China) according to the manufacturer's instructions. Cells were plated at density of 3×10^5^ cells pre 12-well or 6-well plate, and were grown to 60%-70% confluence prior to transfection.

### Luciferase reporter gene assays

The Huh7 cells were co-transfected with plasmid and the promoter. The cell lysates were prepared, and luciferase activity was measured in each sample 48 hours after transfection using the dual-luciferase reporter assay system (Promega Corporation). An internal control was a Renilla lucierase reporter vector pRL-TK. The luciferase assay substrates (80μl) and cell lystates (20μl) were mixed, and then placed at room temperature for 30 minutes. The fluorescence intensity was detected using a luminometer (Promega). All assays were preformed in triplicate, expressed as mean±standard error of the mean relative to the vector control samples, which was set at 100%.

### Quantitative real-time reverse transcription polymerase chain reaction assay

Total RNAs from paired liver tissues and Huh7 cell lines collected at 0, 12, 24 and 48 hours after transfection by pCMV-HBV-1.2, pCMV-HBV-1.2*7 or pCMV-HBX were extracted with Trizol® reagent (Invitrogen, Life Technologies) according to manufacturer's instructions. cDNAs for the detection of RKIP was synthesized using the cDNA First-Strand Synthesis kit (Toyobo, Osaka, Japan). The primers were synthesized by GenScript (Nanjing) Company, which were listed in Table 1. To determine the levels of RKIP mRNA expression, real-time-PCR reactions were performed on an iQ^TM^5 PCR instrument with the following thermal cycle conditions: 40 cycles of 30s at 94°C, 30s at 55°C and 1min at 72°C. Glyceraldehyde-3-phosphate dehydrogenase (GAPDH) was measured by the same method and used as an internal control for normalization.

**Table 1:** Primers Sequence used in the present study for PCR

Assay	Name	Sequence (5′→3′)
Promoter cloning	P1-F1	AGGTCAGGTGAAGCGTCGCGCA
	P2-F2	TTGCAACACTGTCAGGCGTTGC
	P3-F3	ACGGGACAACGTTCATGAA
	P4-F4	TAAGCCGCTTACTCGTTAT
	P5-F5	CAGGTTCGCATGCCAAGCATC
	R	ATGCCCTAACGTCGACGTCCA
Chip	AP1-F	AGGTCAGGTGAAGCGCGTGGT
	AP1-R	CGTTGCAACACTGTCGGCA
Real-Time PCR	RKIP-F	CCGGACAACGTTTATATGACCTG
	RKIP-R	TAGCCGCTTACATCGTTACG
	GAPDH-F	CAGGTTCGCATGCCAGCATCAA
	GAPDH-R	AGCCCGTAACGTCCCACGTCT
Mutagenesis	Mutant-F	CGTTGCAACACTGCATGGCGG
	Mutant-R	CGGACAACGTACTATGATTGCT

### Western blot analysis

Matched liver cancer and adjacent non-cancerous liver tissues were subjected to western blot analysis. Meanwhile, Huh7 cells were transfected with pCMV-HBV-1.2, pCMV-HBV-1.2*7 and pCMV-HBX. Samples were also collected at 0, 24, 48 and 72 hours after transfection and analyzed by western blot to assess RKIP expression level. Whole tissue and cell lysates were prepared by lysing the samples in ice-cold radio-immunoprecipitation buffer (RIPA), and protein extracts were quantified using the bradford protein assay. Then proteins were separated on 10% SDS-PAGE gels electrophoresis and transferred onto nitrocellulose membranes. The membranes were incubated in 5% nonfat dry milk for 1 hr at room temperature. Next, a rabbit polyclonal antibody against RKIP (abcam Corporation) was diluted at 1:2000 and incubated at 4°C for overnight. The membrane was washed before incubation with 1:5000 diluted horseradish-conjugated goat anti-rabbit secondary antibody for 1 hr at room temperature. After autoradiography acquisition, the membranes were stripped and reprobed with anti-GAPDH antibody to normalize RKIP protein expression. The results were visualized using an ECL detection system and band signals were quantified with related image analysis software.

### RKIP promoter methylation

To investigate the effects of CpG islands methylation within RKIP promoter, we transfected Huh7 cells with pCMV-HBV-1.2 plasmid and pCMV-pblue. After 48 hours, we extracted genomic DNA and the detection of RKIP promoter methylation was finished by BioMiao Biological Technology (Beijing) Company. In addition, the Huh7 cells were co-transfected with pCMV-HBV-1.2 plasmid and the promoter of RKIP, then different concentrations of 5-aza-CdR were added. Luciferase activity was measured in each sample 48 hours later. We also assessed RKIP mRNA and protein expression levels.

### Chromatin immunoprecipitation (ChIP)

To further confirm whether the RKIP promoter region had acitivitor protein 1 core site, Huh 7 cells were collected and treated with 1% formaldehyde. Chromatin immunoprecipitation (ChIP) assay was performed using Millipore 17-371 EZ CHIP KIT directed by the manufacturer's instructions. AP1 protein antibody was purchased from abcam company. Primers were designed according to the AP1 binding sites sequences and were listed in Table 1.

### Co-Immunoprecipitation

To investigate the interactive effects of HBX and AP1, Hhu7 cells were collected after transfection and lysed in RIPA buffer, then treated with 20μl proteinase inhibitor cocktail. Cell lysates were sonicated using ultrasonic to interrupt the large pieces of genome DNA, then 2ug antibody was mixed. IgG was used as a negative control. Immunoprecipitates were washed and resuspended sequentially with 30ul 1×SDS loading buffer, then boiled at 100°C for 5 minutes to fully elute and denature the proteins. After centrifuging at 12000rpm for 5 minutes, the supernatant was detected in SDS-PAGE.

### Statistical analysis

All statistical analyses were conducted using SPSS version 17.0 software. Qualitative data were analyzed using the χ^2^ test, and quantitative data was analyzed using Student's t test. For all tests, the level of significance was set at *P*<0.05.
